# Psychological Wellbeing and Aortic Stiffness: Longitudinal Study

**DOI:** 10.1161/HYPERTENSIONAHA.119.14284

**Published:** 2020-07-13

**Authors:** Ai Ikeda, Andrew Steptoe, Martin Shipley, Ian B. Wilkinson, Carmel M. McEniery, Takeshi Tanigawa, Archana Singh-Manoux, Mika Kivimaki, Eric J. Brunner

**Affiliations:** 1 From the Department of Epidemiology and Public Health, Institute of Epidemiology and Health, Faculty of Population Health Sciences, University College London, United Kingdom (A.I., A.S., M.S., A.S.-M., M.K., E.J.B.); 2 Department of Public Health, Juntendo University Graduate School of Medicine, Tokyo, Japan (A.I., T.T.); 3 Division of Experimental Medicine and Immunotherapeutics, University of Cambridge, United Kingdom (I.B.W., C.M.M.); 4 Université de Paris, Inserm U1153, Epidemiology of Ageing and Neurodegenerative Diseases, France (A.S.-M.).

**Keywords:** aged, association, biological factors, cardiovascular disease, longitudinal studies

## Abstract

This study investigated 2 distinct aspects of positive wellbeing: affective wellbeing and eudaimonia with progression of aortic stiffness, an index of subclinical cardiovascular disease. A total of 4754 participants (mean age 65.3 years, 3466 men, and 1288 women) from the Whitehall II cohort study provided data on affective and eudaimonic wellbeing using subscales from the control, autonomy, self-realization and pleasure-19 questionnaire. Aortic stiffness was measured by aortic pulse wave velocity (PWV) at baseline (2008–2009) and 5 years later (2012–2013). Linear mixed models were used to measure the effect of affective and eudaimonic wellbeing on baseline PWV and 5-year PWV longitudinal change. A 1-SD higher eudaimonic wellbeing was associated with lower baseline PWV in men (β=−0.100 m/s [95% CI=−0.169 to −0.032]), independent of social, behavioral, and biological factors. This association persisted over 5 years. No such association was found in women (β=−0.029 m/s [95% CI=−0.126 to 0.069]). We did not find any association of positive wellbeing with change in PWV over time in either men or women. In older men, higher levels of eudaimonic wellbeing were associated with lower long-term levels of arterial stiffness. These findings support the notion that the pattern of association between positive wellbeing and cardiovascular health outcomes involves eudaimonic rather than affective wellbeing and is sex-specific.

## Abstract 2

Supplemental Digital Content is available in the text.

Numerous studies have documented the role of negative emotions in the development of cardiovascular disease (CVD).^1^ There has also been a growing interest in the study of positive emotional factors such as happiness and psychological wellbeing in relation to cardiovascular health and disease.^2,3^ Emotional wellbeing derived from items on Center for Epidemiological Studies Depression Scale (CES-D) was associated with reduced risk of stroke, in a 6-year follow-up.^4^ Positive affect was also associated with lower 10-year incidence of coronary heart disease.^5^ Furthermore, studies have linked positive wellbeing to lower levels of individual cardiovascular risk factors (ie, neuroendocrinological, cardiometabolic, and inflammatory)^3^; however, it is unclear whether positive wellbeing may protect against aortic stiffness over time.

The pathways through which positive wellbeing reduces risk of CVD have not been completely elucidated. Plausible explanations include promoting adaptive physiological functioning (ie, lower systemic inflammation, balanced hypothalamic-pituitary-adrenal axis activation, better cardiac autonomic control), motivating better health behaviors (ie, medication adherence, nonsmoking, moderate alcohol use, greater physical activity, better dietary patterns), and buffering the detrimental effects of stress on health.^2,3,6^

Two distinct aspects of positive wellbeing have been identified: affective wellbeing, characterized by feelings of happiness and pleasure; and eudaimonia or flourishing, related to functioning in life such as thriving, the realization of human potential, autonomy, control over one’s destiny, and purposeful engagement with life.^7^ Although affective and eudaimonic wellbeing may share underlying psychological mechanisms,^8^ the conceptual distinction is important because the 2 constructs may have different biological underpinnings (eg, eudaimonic wellbeing may be more strongly related to diurnal cortisol secretion than affective wellbeing).^9,10^ Moreover, previous researches predominantly examined affective wellbeing in the development of CVD,^2,3^ and there is some evidence that eudaimonic wellbeing is also associated with cardiovascular-related biomarkers.^11–13^ There has been only one prospective study based on a small cohort of women that examined eudaimonic wellbeing in relation to markers of atherosclerosis. This study, based on the Healthy Women Study (n=155; mean age was 65.1 years old), found higher levels of mastery and life purpose to be associated with lower aortic, but not coronary calcification.^14^

The control, autonomy, self-realization and pleasure-19 (CASP-19) is a measure of the quality of life, developed initially for people aged 65 to 75. It captures 4 domains of wellbeing: pleasure or enjoyment of life, control, autonomy, and self-realization^15^ and has been widely used in longitudinal population cohort studies such as the English Longitudinal Study of Ageing and the US Health and Retirement Study. Items of the pleasure domain are used as a measure of affective wellbeing,^16^ while the other 3 domains constitute an index of eudaimonic wellbeing.^17^ The inclusion of the CASP-19 in the Whitehall study provided an opportunity to assess both these components.

Aortic pulse wave velocity (PWV), in turn, measures arterial stiffness involved in cardiovascular pathogenesis, with higher PWV being associated with increased CVD risk.^18^ Following the methods of Vanhoutte,^19^ we divided CASP-19 into 2 composite scales (ie, affective and eudaimonic wellbeing) and examined the relation of affective and eudaimonic wellbeing with PWV. We hypothesized that affective and eudaimonic wellbeing would be associated with (1) lower PWV at baseline (2007–2009) and (2) slower age-related progression of aortic stiffness, indicated by smaller increases in PWV over time (changes from 2007–2009 to 2012–2013). We speculated that these associations might be affected by sex because of sex differences in the biological correlates of positive wellbeing.^13^

## Methods

Data, analytic methods, and study materials of this study are available from the corresponding author on reasonable request (data sharing policy: https://www.ucl.ac.uk/epidemiology-health-care/research/epidemiology-and-public-health/research/whitehall-ii/).

### Study Population

We used data from the ongoing Whitehall II cohort study. Briefly, 10 308 male and female London-based civil servants, aged 35 to 55 years, were recruited to the study in 1985 with a response rate of 73%.^20^ Participants have since been followed up with questionnaire surveys and clinical examinations every 4 to 5 years. Written informed consent was obtained from all participants at each follow-up clinical examinations.

**Figure 1.**
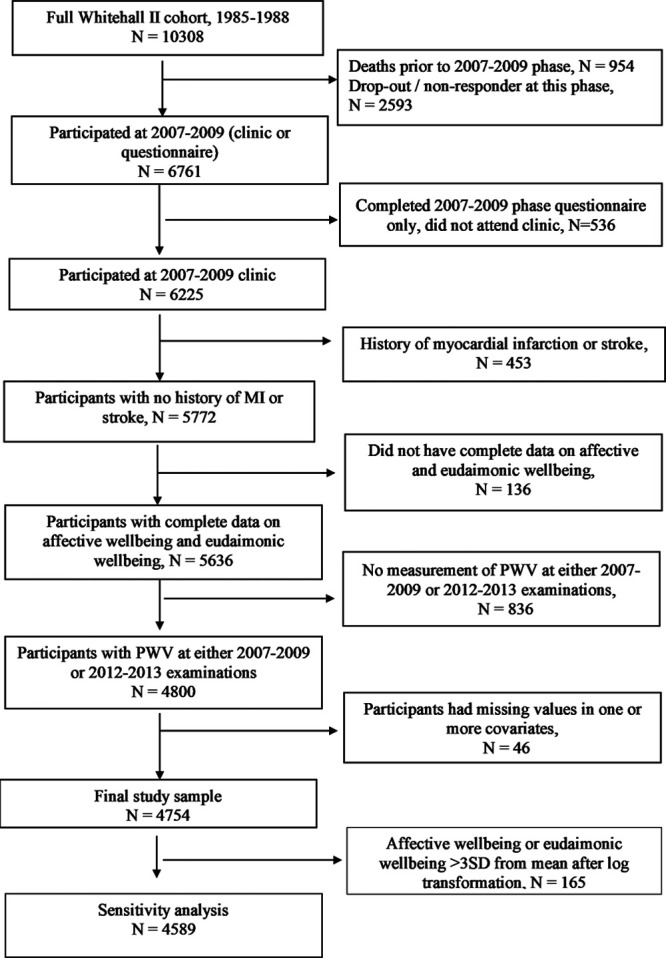
Cohort flowchart. MI indicates myocardial infarction; and PWV, pulse wave velocity.

Eligibility for the present study required continued participation as of the time when positive wellbeing measures were collected from 2007 to 2009 (n=6761). Among the 5772 participants who attended the clinical examination and did not have a history of myocardial infarction (MI) or stroke before 2008 to 2009, a total of 4754 (men=3466, women=1288) had usable positive wellbeing values, at least one measurement of PWV measure at 2007 to 2009 or 2012 to 2013 clinical examination and had no missing values in any covariates, were included in the present study (Figure 1). Sixty-eight percent of participants had PWV measures at both time points (n=3241). Ethical approval was obtained from the National Health Service National Research Ethics Service and the local Research Ethics Committee.

### Assessment of PWV

PWV was assessed between the carotid and femoral sites using applanation tonometry (SphygmoCor, Atcor Medical, Sydney, Australia). The time difference between the peak of the R-wave on ECG and the foot of the pulse waveform captured by the tonometer at the site of the carotid pulse determined the blood transmission time between the heart and carotid pulse. Blood transmission time between the heart and femoral pulse was measured by the same method. The transit time was defined as the difference between the heart-carotid and heart-femoral blood transmission times. Path length was determined with a tape measure by subtracting the carotid-sternal notch distance from the femoral-sternal notch distance. PWV (m/s) was calculated by dividing the path length over the transit time.^21^ In each participant, PWV was measured twice and if the difference in velocity between the 2 measurements was larger than 0.5 m/s, a third measurement was taken. The average of the measurements was used in the analysis.

### Assessment of Positive Wellbeing

We used the CASP-19 questionnaire to index positive wellbeing.^22^ Affective wellbeing was measured with pleasure in life subscale from the CASP-19 quality of life instrument.^22^ This consists of 5 items (eg, I enjoy things that I do, I enjoy being in the company of others), each of which is assessed on a 4-point scale from 1=never to 4=often. The ratings were averaged and scaled from 5 (lowest) to 20 (highest). The Cronbach α in this population was 0.78. Eudaimonic wellbeing was assessed with the remaining 14 items of the CASP-19, which measured control (eg, I feel that what happens to me is out of my control, reverse coded), autonomy (eg, I feel that I can please myself what I do), personal growth (eg, I choose to do things that have never done before), and self-realization (eg, I feel satisfied with the way my life has turned out) that make up psychological wellbeing in Ryff’s taxonomy.^23^ The Cronbach α in this population was 0.85, indicating good internal consistency. Scores could range from 14 to 56. A previous study that tested a 2-factor solution, isolating pleasure from control, autonomy, and self-realization showed a relatively good model fit (root mean square error of approximation=0.08; comparative fit index=0.96; Tucker-Lewis index 0=0.95).^19^ The wellbeing measures were natural log-transformed to improve the normality of the data distributions.

### Measurement of Other Covariates

Medical history (MI, stroke, hypertension, and diabetes mellitus), measures of biomarkers, and information from completion of the questionnaire, including their lifestyle (smoking and alcohol drinking) and psychosocial factors (employment grade and depression) were updated in each clinical examination in 2007 to 2009 and 2012 to 2013. Depression was measured using CES-D.^24^ CES-D consists of 20 items and summing of all items for each participant provides the total score; this can range between 0 and 60. British Civil Service grade of employment was used as a comprehensive measure of socioeconomic status that reflects education, occupational status, and income, composed of a 5-level variable.^20^ Clinical history of MI, stroke, hypertension, and diabetes mellitus were ascertained by self-reported doctor diagnosis on questionnaire surveys. Medication use was examined by questionnaire and the assessment of medications brought by the participant to the clinic visit. Height and weight were recorded in light clothing for the calculation of body mass index (BMI). After a 5-minute rest period, systolic blood pressure was measured twice using an automated UA-779 digital monitor. Mean arterial pressure was calculated as diastolic pressure plus one-third of the pulse pressure. Resting heart rate (HR) was measured via ECG with participants in the supine position. Serum total cholesterol was determined on a Roche P Modular platform after an overnight fast or at least 4 hours after a fat-free breakfast.

### Statistical Analysis

ANOVA to compare mean values and χ^2^ tests to compare proportions were used in the descriptive analyses of the baseline characteristics of men and women. Linear mixed models were used to measure the effect of positive wellbeing (per 1-SD higher on the log-transformed scale) on baseline PWV (2007–2009) and PWV longitudinal change between 2007 to 2009 and 2012 to 2013. These models use all available data over the follow-up, handle differences in length of follow-up, and account for correlation between repeated measures on the same individual. The linear mixed models included a term for time (individual follow-up in years divided by 5, to yield effects on change in PWV over 5 years). The main effect estimates the effect of positive wellbeing on PWV at baseline, whereas positive wellbeing × time interaction term estimates the mean difference in the 5-year change in PWV.

Covariates in all multivariable analyses were age (years), ethnicity (white or nonwhite), employment grade (low-, middle-, or high-grade), CES-D (log-transformed), BMI (kg/m^2^), current smoker (yes/no), alcohol intake in the past week (yes/no), total cholesterol (mmol/L), mean arterial pressure (mm Hg), HR (bpm), ever had hormone replacement therapy (yes/no), hypertension medication use (yes/no), and history of diabetes mellitus (yes/no). The above covariates were measured closest in time to when PWV was measured. Forty-six participants(1%) had missing values in one or more covariates and were excluded from all analyses. These participants were more likely to be female, nonwhite, have low employment grade and less likely to drink alcohol. In analysis, all these factors were controlled. Sensitivity analyses excluding participants whose log-transformed wellbeing values were greater than 3-SD from the mean were conducted.

All analyses were stratified by sex and were conducted using the SAS statistical package version 9.1 (SAS Institute Inc, Cary, NC). Statistical significance was defined as *P*<0.05.

## Results

The 4754 participants in the study sample were younger, contained smaller percentages of women and nonwhites and generally had a better risk profile than the 1081 participants with no history of MI or stroke who attended the 2007 to 2009 screening clinic, but who were excluded from the final sample (Table I in the Data Supplement).

The characteristics of men and women are presented in Table 1. The proportion who were nonwhite, nondrinkers, and of a lower grade of employment was higher in women than men. Women were more likely to have higher levels of depressive symptoms measured by CES-D, BMI, HR, and cholesterol compared with men. However, the levels of systolic blood pressure and mean arterial pressure were lower in women than men. The Spearman correlation coefficient between affective and eudaimonic wellbeing was 0.62 (*P*<0.001) for men and 0.66 (*P*<0.001) for women (not listed in Table).

**Table 1.**
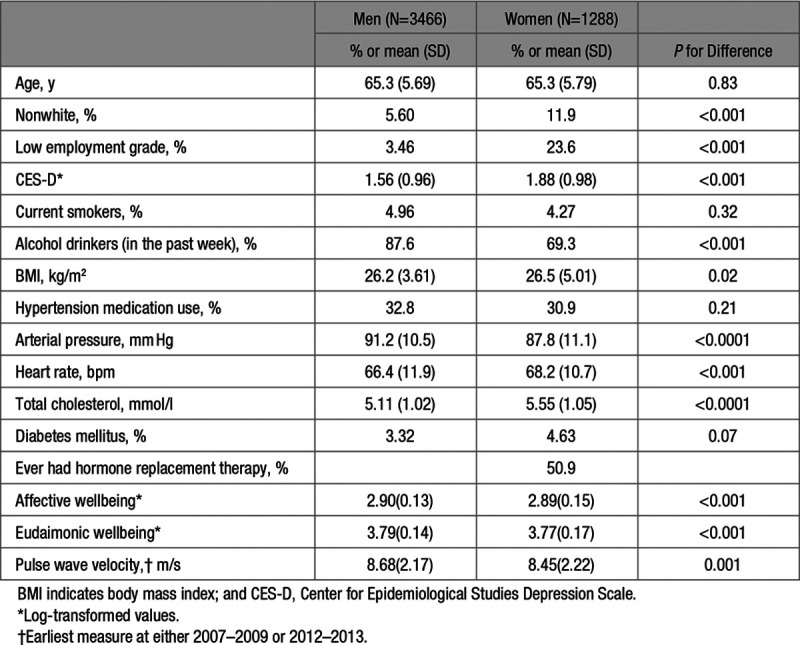
Baseline Characteristics (2007–2009) According to Sex

The age-adjusted mean PWV changed from 8.50 m/s (SE=0.04) at baseline to 9.22 m/s (SE=0.04) 5 years later for men and 8.20 m/s (SE=0.06) at baseline to 8.85 m/s (SE=0.06) for women. The sex-specific multivariable-adjusted mean differences in PWV associated with a 1-SD difference in positive wellbeing at the baseline (2007–2009) are reported in Table 2. A significant association between the baseline PWV and a 1-SD difference in eudaimonic wellbeing (β=−0.100 m/s [95% CI=−0.169 to −0.032]) was found in men (model 2). This relationship was independent of age, ethnicity, employment grade, alcohol intake, smoking status, depression, hypertension medication use, diabetes mellitus, and the levels of cholesterol, mean arterial pressure, and HR at PWV measurement. However, no associations between positive wellbeing and PWV were evident in women. In sensitivity analysis, we repeated all analyses excluding participants who have wellbeing >3-SD from the mean (on the log-transformed scale; Table II in the Data Supplement). The exclusion did not change the results materially.

**Table 2.**
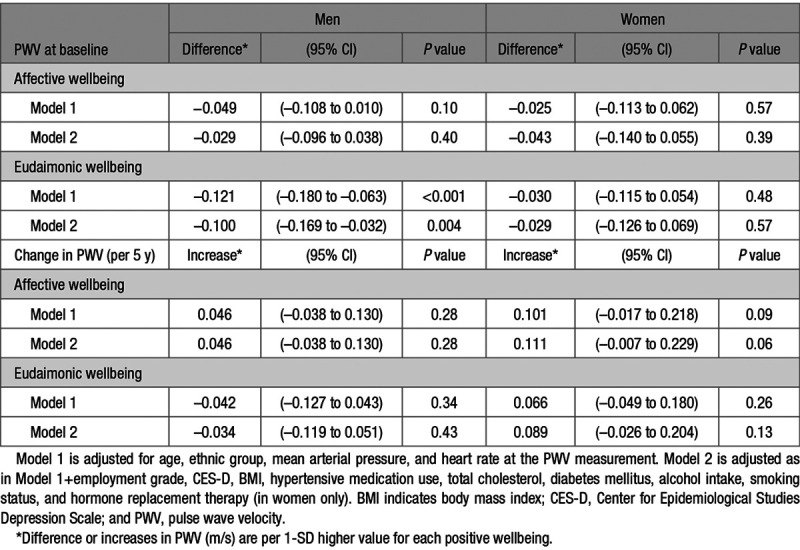
Association of Positive Wellbeing With Baseline PWV (2007–2009) and 5-Year Progression of PWV Controlling for Demographic, Behavioral, and Biomedical Factors

**Figure 2.**
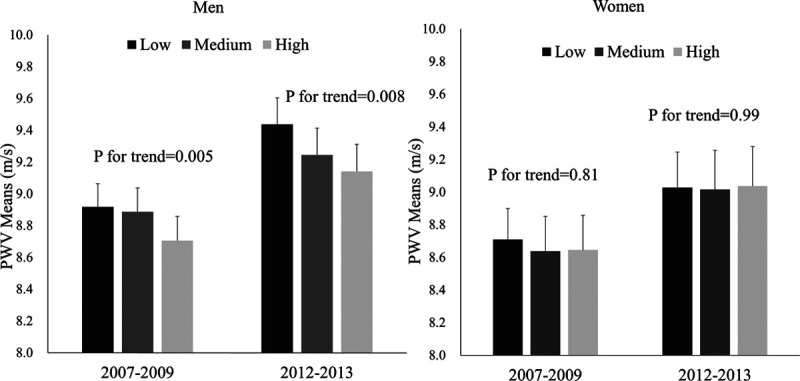
Association between eudaimonic wellbeing and pulse wave velocity (PWV). Eudaimonic wellbeing is divided into tertiles. Error bars show the SEM. PWV means are adjusted for age, ethnic group, mean arterial pressure, and heart rate at the PWV measurement.

The sex-specific multivariable-adjusted mean difference in the 5-year change in PWV associated with a 1-SD higher positive wellbeing is also presented in Table 2. We did not find any association of positive wellbeing with change in PWV over time in either men or women. As there was no evidence that eudaimonic wellbeing was associated with rate of change in PWV over time, the association between the higher levels of eudaimonic wellbeing and lower levels of PWV at the baseline persisted 5 years later in men (Figure 2).

To assess the role of each factor in the association between eudaimonic wellbeing and PWV, Table III in the Data Supplement shows the association between the tertiles of eudaimonic wellbeing and baseline characteristics. Persons, both men and women, with higher eudaimonic wellbeing were more likely to be white, of higher employment grade, be an alcohol-drinker, have a lower level of depression score, and have a higher affective wellbeing. Moreover, men with higher eudaimonic wellbeing were also more likely to have a lower level of BMI and HR and fewer used hypertension medication; similar patterns were not found in women.

## Discussion

In this longitudinal analysis of older adults, we found a persistent association between the eudaimonic wellbeing and lower aortic stiffness in men but no relation with rate of change in aortic stiffness over 5 years. Men reporting high eudaimonic wellbeing had lower PWV, indicative of lower aortic stiffness and lower CVD risk. These findings were maintained after adjusting for an array of social, behavioral, and biologic factors, suggesting that other mechanisms linking positive wellbeing with these biological alterations will need to be considered. We found no significant associations of affective wellbeing with PWV and no associations among women.

Few previous studies have examined the association between arteriosclerosis and positive wellbeing. To our knowledge, this is the first prospective cohort study to examine the direct effects of positive wellbeing on change in PWV, using repeated measures, over a period of years. Our finding was consistent with previous work that has found an association between mastery (ie, perception of his/her capacity to control over life circumstances) and an increased risk of CVD mortality.^25^ Moreover, evidence from the INTERHEART case-control study demonstrated that the perceived ability to control life circumstances was associated with reduced risk of first MI.^26^ Japanese cohort studies found that ikigai (ie, a sense of purpose of life) had a protective effect against CVD mortality.^27,28^ A recent meta-analysis has reported that the hazard ratio (95% CI) per SD change in log_e_-transformed PWV was 1.30 (1.18–1.43) and 1.28 (1.15–1.43) for the risk of total CVD events and cardiovascular mortality, respectively.^18^ Combining these results with previous studies may give some insight into the potential functional significance of our findings in relation to aortic stiffness underlying the association between eudaimonic wellbeing and CVD development or progression.

No association between affective wellbeing and reduced risk of aortic stiffness was found in our present study. Affective wellbeing has not consistently been associated with incident coronary heart disease.^5,29^ Moreover, Boehm et al^30^ found life satisfaction was not associated with MI and coronary death in the Whitehall II study cohort. Similarly, unhappiness was not associated with mortality from ischemic heart disease among 719 671 women aged 50 to 69 years who participated in the Million Women Study.^31^ However, Boehm et al^32^ found that psychological wellbeing predicted reduced cardiovascular mortality in the English Longitudinal Study of Ageing. However, considering the limited set of studies to date and inconsistent association with coronary heart disease, the effect of affective wellbeing on cardiovascular biomarkers is still not entirely clear. Moreover, Matthews et al^14^ found an association between aortic calcification and life satisfaction measured by Life Engagement Test in women. The life engagement test is designed to measure purpose in life, while the pleasure domain of CASP-19 is designed to evaluate self-reflective levels of pleasure or enjoyment of person’s lives. These differences in the measures of affective wellbeing may be responsible in part for this inconsistency.

The potential pathways linking between positive wellbeing and CVD may involve at least 3 processes: (1) direct effects on biological mechanisms; (2) indirect effect through healthy lifestyles and behavior; and (3) promoting other psychosocial resources known to protect health or buffer cardiotoxic stressful life experiences.^33^ While eudaimonic wellbeing scores were lower among men with biological (higher BMI, HR, and preexisting hypertension), behavioral (higher BMI), and psychosocial factors (lower employment grade) known to contribute aortic stiffness progression,^34–37^ the association was independent of these risk factors in the present study. This association was also independent of negative states such as depressed mood that lie at the opposite pole to positive wellbeing.^38^ More dynamic stress-related biological processes such as increased inflammation and variability in autonomic tone may contribute to the associations observed here.^39^

The associations between eudaimonic wellbeing and PWV differed by sex in the present study. A previous study has reported that quality of life (CASP-19) declined over time in patients with coronary heart disease who, as a result of the disease, have experienced loss of control and autonomy.^40^ The decline in quality of life was more evident in men than women, and the lower autonomy associated with coronary heart disease events was also found in men compared with women.^40^ It has been argued that women may generally have more effective coping strategies for managing stressful experiences than men.^41^ In previous studies, the inverse relationships between cardiometabolic function (ie, HR and central adiposity) and positive wellbeing have been found in men but not in women.^13,42^ However, inverse associations between wellbeing and inflammatory makers (ie, plasma C-reactive protein, interleukin 6, and high-density lipoprotein cholesterol) have been found in women rather than men.^13,43^ In the present study, eudaimonic wellbeing in men, but not in women, was associated with a favorable atherosclerosis risk profile, characterized by lower BMI and HR and lower hypertension medication use. However, our present findings were maintained after adjusting for an array of social, behavioral, and biologic factors, suggesting that other mechanisms linking wellbeing with these biological alterations may explain the observed sex differences.

Strengths of the study include the use of a well-characterized cohort of men and women who have been followed over a long period of time with a wide range of biological variables, measurement of both affective wellbeing and eudaimonic wellbeing, and extensive statistical control for sociodemographic, behavioral, and health covariates. A measure of PWV was obtained twice for 70% of participants. However, the cohort is drawn from a white-collar population, so further research is needed to assess the possibility that stronger associations between positive wellbeing and PWV are present in more diverse populations. Moreover, although we adjusted for various possible confounding factors in the current study, there is a possibility of residual confounding by unmeasured variables, such as genetic factors,^44,45^ that may influence the CVD causality cascade. In the present analysis, neither affective or eudaimonic wellbeing was associated with the rate of change in PWV over time. These findings do not lend strong support for a causal effect of psychological wellbeing on atherosclerotic processes. However, eudaimonic wellbeing demonstrated consistent associations with PWV across the 2-time points. This finding is consistent with the explanation that the rate of change in trajectories was set earlier in life, either at younger ages or at an earlier stage with regard to the atherosclerotic processes. This suggests that the maintenance of relative differences in PWV levels will continue until older ages, but differences in rates of change may not. Furthermore, the measure of eudaimonic wellbeing encompassed several aspects of the construct as defined by Ryff and Keyes^23^ but did not include other aspects such as positive relationships that have been enumerated in other research. Previous research has found higher scores on positive relationships with others for women, compared with men.^46,47^ This may have attenuated the strength of associations between eudaimonic wellbeing and aortic stiffness in women. Last, we found a moderately high correlation (0.62 in men and 0.66 in women) between affective and eudaimonic wellbeing. This is in agreement with previously reported correlations between affective wellbeing (or pleasure) and eudaimonic domains (ie, autonomy, control, and self-realization) which range from 0.4 to 0.6.^15^ Despite this, the associations between affective and eudaimonic wellbeing and PWV were little changed after mutual adjustment. The link between eudaimonic wellbeing and PWV was particularly robust attenuating by <5% in adjusted models.

## Perspective

We found that higher eudaimonic wellbeing was associated with lower PWV at baseline in men. The association persisted over 5 years and remained after adjusting for multiple biological, behavioral, and psychosocial factors. These findings lend weight to the notion that the pattern of association between positive wellbeing and cardiovascular health outcomes involves eudaimonic rather than affective wellbeing and is sex-specific. Eudaimonic wellbeing has been linked with a number of other favorable physiological and behavioral outcomes at older ages,^48^ and the present study endorses its relevance to CVD as well.

## Acknowledgments

We thank all of the participating civil service departments and their welfare, personnel, and establishment officers; the British Occupational Health and Safety Agency; the British Council of Civil Service Unions; all participating civil servants in the Whitehall II study; and all members of the Whitehall II study team. The Whitehall II Study team comprises research scientists, statisticians, study coordinators, nurses, data managers, administrative assistants, and data entry staff, who make the study possible.

## Sources of Funding

The Whitehall II study is supported by grants from the British Heart Foundation (RG/16/11/32334), British Medical Research Council (MR/S011676, R24227), and the US National Institute on Aging (RF1AG062553, R01AG034454). A. Ikeda is supported by JSPS KAKENHI Grant Number JP17KK0175. E.J. Brunner is supported by the British Heart Foundation (RG/16/11/32334) and the European Commission (FP7 project no. 613598). M. Kivimäki is supported by NordForsk, the Nordic Programme on Health and Welfare, Academy of Finland (311492), and Helsinki Institute of Life Science. Funding sources did not have a role in the design and conduct of the study, the collection, management, analysis, and interpretation of the data or the preparation, review, approval, or decision to submit the manuscript.

## Disclosures

None.

## Supplementary Material



## Novelty and Significance

### What Is New?

- This study investigated 2 distinct aspects of positive wellbeing: affective wellbeing and eudaimonia with progression of aortic stiffness, an index of subclinical cardiovascular disease. To our knowledge, this is the first prospective cohort study to examine the direct effects of positive wellbeing on change in aortic stiffness over a period of years.

### What Is Relevant?

- Eudaimonic wellbeing has been linked with a number of other favorable physiological and behavioral outcomes at older ages, and the present study also endorses its relevance to cardiovascular disease.

### Summary

In older men, higher levels of eudaimonic wellbeing were associated with lower long-term levels of arterial stiffness. We found no significant associations of affective wellbeing with PWV and no associations among women. The pattern of association between positive wellbeing and cardiovascular health outcomes involves eudaimonic rather than affective wellbeing and is sex-specific.
