# Supplementary figures and images for: Psychological Wellbeing and Aortic Stiffness: Longitudinal Study
Source: Hypertension. 2020 Jul 13;76(3):675–82. doi: 10.1161/HYPERTENSIONAHA.119.14284 (PMC7418936; doi:10.1161/HYPERTENSIONAHA.119.14284)

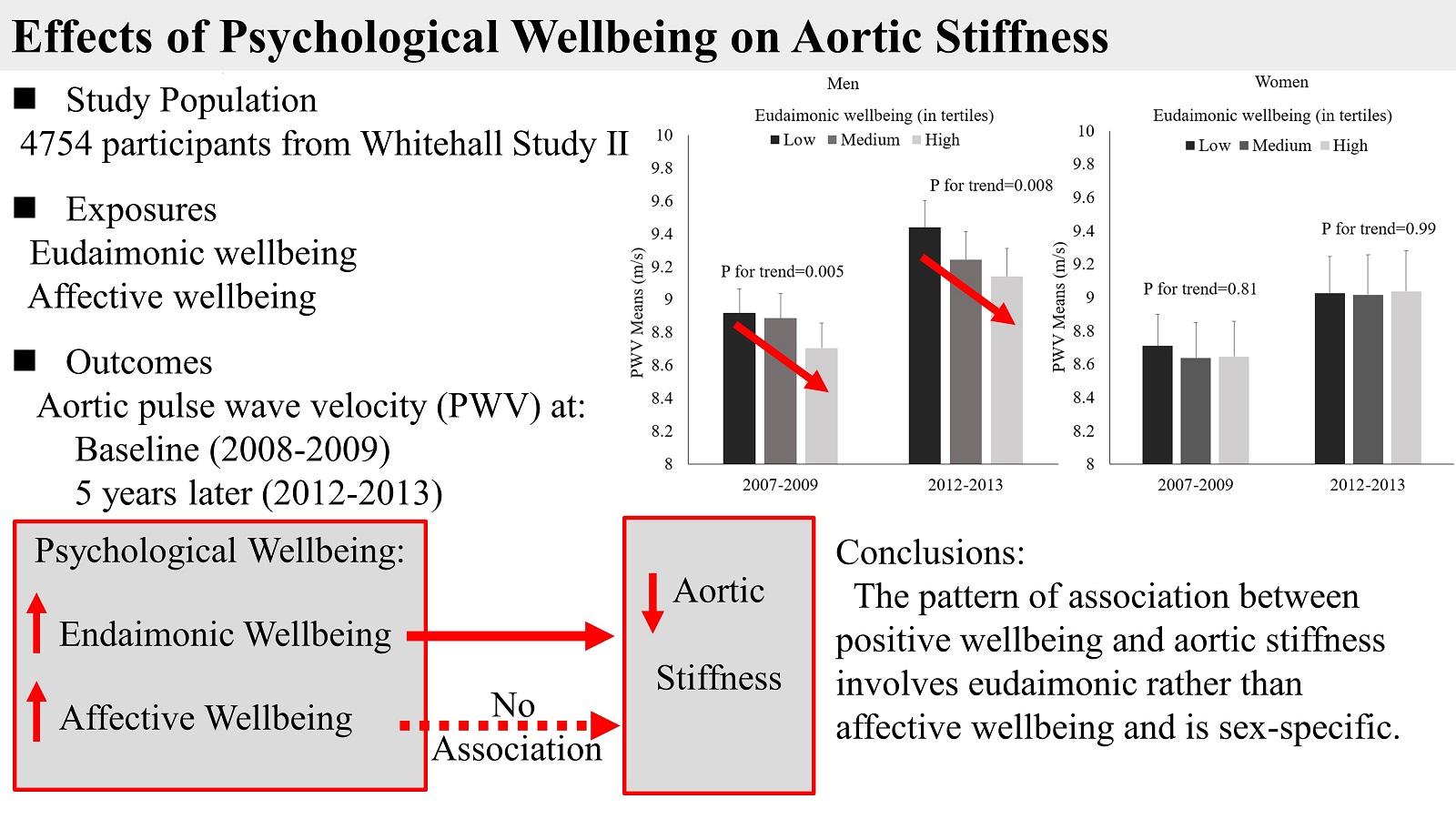

Supplement: Supplementary file 1 [file hyp-76-0675-s001.jpg]
