# Supplementary material for: Psychological Wellbeing and Aortic Stiffness: Longitudinal Study
Source: Hypertension. 2020 Jul 13;76(3):675–82. doi: 10.1161/HYPERTENSIONAHA.119.14284 (PMC7418936; doi:10.1161/HYPERTENSIONAHA.119.14284)
Supplement: Supplementary file 2 [file hyp-76-0675-s002.docx]

**Psychological wellbeing and aortic stiffness: longitudinal study**

Ai Ikeda, PhD^1,2^, Andrew Steptoe, DPhil^1^, Martin Shipley, MSc^1^, Ian B Wilkinson^3^, Carmel M McEniery^3^, Takeshi Tanigawa ^2^, MD, Archana Singh-Manoux, PhD^1,4^, Mika Kivimaki, PhD^1^, Eric J. Brunner, PhD^1^

^1^ Department of Epidemiology and Public Health, Institute of Epidemiology and Health, Faculty of Population Health Sciences, University College London, London, UK

^2^ Department of Public Health, Juntendo University Graduate School of Medicine, Tokyo, Japan

^3^ Division of Experimental Medicine and Immunotherapeutics, University of Cambridge, UK

^4^ Université de Paris, Inserm U1153, Epidemiology of Ageing & Neurodegenerative diseases, Paris, France

**Short title:** Psychological wellbeing and pulse wave velocity

**Correspondence and Reprint Requests:**

Eric Brunner, PhD

Department of Epidemiology and Public Health, University College London
1-19 Torrington Place, London, WC1E 7HB United Kingdom.

Phone:+44 20 7679 1689 Email:[e.brunner@ucl.ac.uk](mailto:e.brunner@ucl.ac.uk)

Table S1: Comparison of characteristics of 5772 participants who attended the screening clinic in 2007-2009, according to whether they are in the final study sample.

|  | Excluded  (N= 1018) |  | Included  (N=4754) |  |
| --- | --- | --- | --- | --- |
|  | % or Mean (SD) |  | % or Mean (SD) | P for difference |
| Age, year | 67.2 (6.21) |  | 65.3 (5.71) | <0.001 |
| Female, % | 40.0 |  | 27.1 | <0.001 |
| Non-white, % | 9.43 |  | 7.30 | 0.02 |
| Low employment grade, % | 16.4 |  | 8.92 | <0.001 |
| CES-D^*^ | 1.87 (0.97) |  | 1.65 (0.98) | <0.001 |
| Current smokers, % | 7.19 |  | 4.77 | 0.002 |
| Alcohol drinkers  (in the past week), % | 73.0 |  | 82.6 | <0.001 |
| BMI, (kg/m2) | 28.6 (5.50) |  | 26.3 (4.04) | <0.001 |
| Hypertension medication use, % | 42.7 |  | 32.3 | <0.001 |
| Arterial pressure, mmHg | 91.9 (11.3) |  | 90.3 (10.7) | 0.005 |
| Heart rate, bpm | 67.2 (13.2) |  | 66.9 (11.6) | 0.45 |
| Total cholesterol, mmol/l | 5.39 (1.21) |  | 5.23 (1.05) | <0.001 |
| Diabetes, % | 5.82 |  | 3.62 | 0.001 |
| Ever had hormone  replacement therapy, % | 20.0 |  | 13.8 | <0.001 |

^*^Log-transformed value

Table S2. Association of positive wellbeing with baseline PWV (2007-2009) and 5-year progression of PWV controlling for demographic, behavioral and biomedical factors after removing extreme values (>3SD from the means).

|  |  |  | Men |  |  |  | Women |  |
| --- | --- | --- | --- | --- | --- | --- | --- | --- |
| PWV at Baseline | | Difference^*^ | (95% CI) | P-value |  | Difference^*^ | (95% CI) | P-value |
| Affective Wellbeing | |  |  |  |  |  |  |  |
|  | Model 1 | -0.036 | (-0.114,0.043) | 0.37 |  | 0.008 | (-0.114,0.130) | 0.90 |
|  | Model 2 | -0.024 | (-0.111,0.063) | 0.59 |  | 0.001 | (-0.133,0.135) | 0.99 |
| Eudaimonic Wellbeing | |  |  |  |  |  |  |  |
|  | Model 1 | -0.122 | (-0.189,-0.056) | <0.001 |  | -0.024 | (-0.130,0.082) | 0.65 |
|  | Model 2 | -0.103 | (-0.179,-0.026) | 0.01 |  | -0.012 | (-0.131,0.108) | 0.85 |
| Change in PWV (per 5-years) | | Increase^*^ | (95% CI) | P-value |  | Increase^*^ | (95% CI) | P-value |
| Affective Wellbeing | |  |  |  |  |  |  |  |
|  | Model 1 | 0.095 | (-0.018,0.208) | 0.10 |  | 0.038 | (-0.129,0.205) | 0.65 |
|  | Model 2 | 0.093 | (-0.020,0.207) | 0.11 |  | 0.044 | (-0.123,0.211) | 0.61 |
| Eudaimonic Wellbeing | |  |  |  |  |  |  |  |
|  | Model 1 | -0.055 | (-0.152,0.042) | 0.27 |  | -0.019 | (-0.162,0.125) | 0.80 |
|  | Model 2 | -0.046 | (-0.143,0.051) | 0.35 |  | 0.005 | (-0.140,0.149) | 0.95 |

Model 1 is adjusted for age, ethnic group, mean arterial pressure and heart rate at the pulse wave velocity measurement. Model 2 is adjusted as in Model 1 + employment grade, CES-D, BMI, hypertensive medication use, total cholesterol, diabetes, alcohol intake, smoking status, hormone replacement therapy (in women only). ^*^Difference or increases in PWV (m/s) are per 1SD higher value for each positive wellbeing.

Table S3: Baseline characteristics (2007-2009) according to eudaimonic wellbeing in tertiles.

|  | Men | | | |  | Women | | | |
| --- | --- | --- | --- | --- | --- | --- | --- | --- | --- |
| Tertiles of eudaimonic wellbeing | T1 | T2 | T3 | p-for difference |  | T1 | T2 | T3 | p-for difference |
| Eudaimonic wellbeing^*^ | 3.62(0.12) | 3.81(0.03) | 3.92(0.04) |  |  | 3.60(0.15) | 3.81 (0.03) | 3.93(0.04) |  |
| N | 1137 | 1163 | 1166 |  |  | 485 | 388 | 415 |  |
| Age, year | 65.2(5.90) | 65.7(5.80) | 65.0(5.33) | 0.02 |  | 65.5(5.92) | 65.8(5.80) | 64.5(5.56) | 0.005 |
| Non-white, % | 8.53 | 4.64 | 3.69 | <0.001 |  | 15.3 | 12.4 | 7.47 | 0.001 |
| Low employment grade, % | 5.19 | 2.84 | 2.40 | <0.001 |  | 25.4 | 28.4 | 17.1 | <0.001 |
| CES-D^*^ | 2.28(0.74) | 1.51(0.80) | 0.91(0.81) | <0.001 |  | 2.56(0.73) | 1.77(0.82) | 1.19(0.85) | <0.001 |
| Current smokers, % | 4.93 | 4.82 | 5.15 | 0.93 |  | 4.54 | 4.64 | 3.61 | 0.72 |
| Alcohol drinkers (in the past week), % | 85.1 | 88.9 | 88.6 | 0.01 |  | 64.3 | 66.2 | 78.1 | <0.001 |
| BMI, kg/m^2^ | 26.5(3.88) | 26.1(3.46) | 26.0(3.46) | <0.001 |  | 26.6(5.05) | 26.6(4.91) | 26.3(5.08) | 0.87 |
| Hypertension medication use, % | 35.9 | 34.1 | 28.5 | <0.001 |  | 33.2 | 32.5 | 26.7 | 0.08 |
| Arterial pressure, mmHg | 91.3(10.8) | 91.2(10.2) | 91.1(10.4) | 0.84 |  | 88.2(11.0) | 87.9(10.9) | 87.2(11.3) | 0.41 |
| Heart rate, bpm | 67.3(12.2) | 66.3(12.1) | 65.7(11.5) | 0.005 |  | 68.9(10.9) | 68.0(10.4) | 67.4(10.7) | 0.09 |
| Total cholesterol, mmol/l | 5.09(1.05) | 5.07(0.98) | 5.18(1.03) | 0.02 |  | 5.49(1.07) | 5.56(1.10) | 5.60(0.98) | 0.28 |
| Diabetes, % | 4.49 | 3.35 | 2.14 | 0.007 |  | 4.95 | 4.64 | 3.61 | 0.61 |
| Ever had hormone replacement therapy, % |  |  |  |  |  | 53.2 | 51.3 | 47.7 | 0.11 |
| Affective wellbeing^*^ | 2.80(0.16) | 2.93(0.08) | 2.97(0.05) | <0.001 |  | 2.78(0.18) | 2.93(0.07) | 2.97(0.05) | <0.001 |
| Pulse wave velocity, m/s^†^ | 8.82(2.36) | 8.75(2.16) | 8.47(1.97) | <0.001 |  | 8.59(2.29) | 8.52(2.29) | 8.23(2.05) | 0.04 |

^*^ Log-transformed values; ^†^earliest measure at either 2007-2009 or 2012-2013.
